# Bioluminescence of the heterotrophic dinoflagellate *Polykrikos kofoidii* Chatton 1914 (Dinophyceae)

**DOI:** 10.1111/jpy.70157

**Published:** 2026-03-31

**Authors:** Michael I. Latz, Dimitri D. Deheyn, Brittany N. Sprecher

**Affiliations:** ^1^ Scripps Institution of Oceanography University of California San Diego La Jolla California USA; ^2^ Department of Neurosciences University of California San Diego School of Medicine La Jolla California USA

**Keywords:** bioluminescence, dinoflagellate, fluorescence, luciferase, luciferin, luciferin binding protein, microscopy

## Abstract

Heterotrophic dinoflagellates are important predators of phytoplankton, and many species produce bioluminescence, which is thought to serve as an antipredator function. The present study investigated the bioluminescence of the heterotrophic dinoflagellate *Polykrikos kofoidii*, an important predator of toxic and bloom‐forming dinoflagellates. Its flashes were slow and dim compared to those of other dinoflagellates but with a similar spectral emission. Based on hyperspectral and laser confocal microscopy, autofluorescence consistent with that of luciferin was distributed throughout the cell, with only a few punctate sources typical of scintillons from other luminescent dinoflagellates. *Polykrikos kofoidii* consumed whole prey, which initially remained intact with measurable autofluorescence of chlorophyll, if plastid‐containing, and luciferin, if luminescent. *Polykrikos kofoidii* encoded a luciferase gene with three conserved catalytic domains with an N‐terminal region that appeared to contain a luciferin‐binding protein‐like motif. In three of the nine publicly available *P*. *kofoidii* transcriptomes, a luciferin‐binding protein with homology to *Noctiluca scintillans* hybrid luciferase‐luciferin binding domain was detected. The slow and dim flashes of *P. kofoidii* resulted in a bioluminescence signature that was distinct from other luminescent dinoflagellates, whereas the diffuse cellular distribution of luciferin fluorescence was unlike the punctate scintillon emission observed in previously studied luminescent dinoflagellates. This suggests that the cellular regulation of luciferin in *P. kofoidii* may differ from that of other dinoflagellates.

**Abbreviations**
ANOVA: analysis of variance
Ca^2+^
: calcium
D1: luciferase catalytic domain 1
D2: luciferase catalytic domain 2
D3: luciferase catalytic domain 3
EDTA: ethylenediaminetetraacetic acid
FSW: (autoclaved) filtered seawater
FWHM: full width at half maximum
GF/F: glass fiber filters
HCl: hydrochloric acid
iTOL: interactive tree of life
ITS: internal transcribed spacer
LBP: luciferin binding protein
*lbp*
: luciferin binding protein gene
*lcf*
: luciferase gene
LCF: luciferase protein
MAFFT: Multiple Alignment using Fast Fourier Transform
ML: maximum likelihood
NA: numerical aperture
PARISS: Prism And Reflector Imaging Spectroscopy System
PCR: polymerase chain reaction
pLDDT: per‐residue structure confidences
SNR: signal‐to‐noise ratio
SRA: sequence read archive
TMSL: total mechanically stimulated bioluminescence
UBAT: underwater bioluminescence assessment tool
WAG: Whelan and Goldman

## INTRODUCTION

Heterotrophic dinoflagellates are important predators of phytoplankton (Sherr & Sherr, 2007; Tillmann, 2004) and include bloom‐forming and toxic dinoflagellates that can form red tides (Jeong, 1999; Jeong et al., 2010). Heterotrophic dinoflagellates can respond rapidly to prey availability due to their high population growth rates (Du Yoo et al., 2015; Strom & Morello, 1998). As such, they can play a critical role in modulating the dynamics of harmful algal blooms including red tides.

Among dinoflagellates, bioluminescent taxa are spread across more than a dozen genera (Haddock et al., 2010; Herring, 1977; Marcinko et al., 2013; Valiadi et al., 2012). The mechanism of dinoflagellate bioluminescence is consistent across all species and is produced in response to mechanical stimuli including flow and contact (Latz, Juhl, et al., 2004; Latz, Nauen, & Rohr, 2004; Rohr et al., 1998; von Dassow et al., 2005), which cause deformation of the plasma membrane (Jalaal et al., 2020; Tesson & Latz, 2015) and activation of a rapid mechanosensing pathway (Eckert, 1965; Latz et al., 2008; Widder & Case, 1981a) involving highly conserved signaling proteins (Lindström et al., 2017). The chemical energy resulting in light emission comes from the oxidation of luciferin, the substrate of the light‐producing reaction, which in dinoflagellates is localized in vesicles called scintillons, the distribution of which can be observed based on luciferin autofluorescence from punctate microsources within the cell (Fritz et al., 1990; Johnson et al., 1985; Latz & Lee, 1995; Widder & Case, 1982). Dinoflagellate bioluminescence, which is usually displayed as short flashes, is considered an important ecological adaptation used for predator defense (Buskey et al., 1983; Cusick & Widder, 2014; Esaias & Curl Jr., 1972; Huang et al., 2024; Marcinko et al., 2013).

One common bioluminescent dinoflagellate, *Polykrikos kofoidii*, is a heterotrophic member of the genus *Polykrikos*, which has a global distribution and is an important member of plankton communities (Manning et al., 2021; Park et al., 2024b). It produces dormant cysts (Attaran‐Fariman et al., 2011; Keskes et al., 2020; Mertens et al., 2020; Morey‐Gaines & Ruse, 1980; Shin et al., 2012; Zonneveld & Dale, 1994) that serve as a resistant life stage that can later reemerge to reestablish populations of swimming cells.

*Polykrikos kofoidii* is one of five described species of *Polykrikos*, which exist as morphologically complex athecate pseudocolonies with two nuclei, regardless of the number of zooids within the pseudocolony (Hoppenrath & Leander, 2007; Tillmann & Hoppenrath, 2013). Prey are captured using extruded nematocysts, harpoon‐like secretory organelles and engulfed whole through the posterior sulcus (Matsuoka et al., 2000). *Polykrikos kofoidii* is strictly heterotrophic and a voracious predator of a wide range of algae including bloom‐forming and toxic dinoflagellates (Du Yoo et al., 2015; Eppley & Harrison, 1974; Jang et al., 2022; Jeong et al., 2001, 2003; Kang et al., 2023; Lee et al., 2017; Matsuyama et al., 1999; You et al., 2020), indicating its key role in the occurrence and fate of algal blooms.

Although *Polykrikos* can be an important contributor to in situ bioluminescence (Cronin et al., 2016; Lapota, Rosenberger, & Lieberman, 1992; Lapota, Young, et al., 1992; Moline et al., 2009; Swift et al., 1995), little is known about the fundamental aspects of its flash emissions, especially concerning which environmental parameters affect their light production and how these parameters compare to other luminous dinoflagellates. The bioluminescence capacity of *P. kofoidii* is less than that of other heterotrophic dinoflagellates (Buskey et al., 1992) and is affected by temperature and salinity (Park et al., 2024a, 2024b).

The objective of the present study was to investigate some fundamental aspects of the bioluminescence of *Polykrikos kofoidii* maintained in laboratory culture, with an emphasis on characterizing its bioluminescence and fluorescence emission spectra, flash kinetics, cellular localization of luciferin, and luciferase‐ and luciferin‐binding proteins, in comparison to those of other bioluminescent dinoflagellates. In addition, the day/night difference in stimulated bioluminescence of *P. kofoidii* was investigated, as the related *P. schwartzii* was observed not to be inhibited by light exposure (Hamman & Seliger, 1972), unlike the heterotrophic dinoflagellates of *Protoperidinium* spp. (Buskey et al., 1992; Li et al., 1996; Tyulkova & Filimonov, 1981) and other autotrophic dinoflagellates wherein bioluminescence is inhibited by light exposure during the dark phase (Esaias et al., 1973; Hamman et al., 1981a, 1981b; Lapota, Young, et al., 1992; Latz & Lee, 1995; Sullivan & Swift, 1994).

The bioluminescence of *Polykrikos kofoidii* was compared to that of other heterotrophic dinoflagellates that were available at the time of this study. *Protoperidinium divergens*, *Pr*. cf. *oblongum*, and *Pr*. cf. *steinii* are important predators of dinoflagellates and other plankton and can reach high concentrations during bloom events (Gribble et al., 2007; Jacobson & Anderson, 1986; Jeong, 1994; Jeong & Latz, 1994; Naustvoll, 2000; Olseng et al., 2002; Raji & Padmavati, 2014; Sathishkumar et al., 2021). All *Protoperidinium* species are pallium feeders, using a tow filament to catch prey, which are then surrounded with a pallium membrane, within which extracellular digestion occurs (Gaines & Taylor, 1984; Jacobson & Anderson, 1986). Their bioluminescence capacity is affected by prey availability (Buskey et al., 1992; Latz & Jeong, 1996).

The bioluminescence of *Polykrikos kofoidii* was also compared to that of some autotrophic dinoflagellates in our culture collection including *Lingulaulax polyedra*, *Ceratocorys horrida, Pyrocystis fusiformis*, and *Py. lunula*. *Lingulaulax polyedra*, previously known as *Lingulodinium polyedra*, is a bloom‐forming autotroph that is responsible for harmful algal blooms along the west coast of North America (Allen, 1946; Gregorio & Pieper, 2000; Gutierrez‐Mejia et al., 2016; Kudela & Cochlan, 2000); the bioluminescence of *L. polyedra* has been well studied for more than 60 years (Hastings & Sweeney, 1957, 1958; Sweeney & Hastings, 1957; Valiadi & Iglesias‐Rodriguez, 2013). *Ceratocorys horrida*, an autotroph that inhabits temperate and tropical waters (Ballantine, 1961; Gaarder, 1954; Hernández‐Becerril, 1988; Hulbert, 1962; Krishnamurthy et al., 1980), has a circadian rhythm of flashing and glow that is similar to that of *L. polyedra* (Latz & Lee, 1995). *Pyrocystis fusiformis* and *Py. lunula* are autotrophs that inhabit oligotrophic and temperate waters and exhibit bright flashes and high bioluminescence capacity (Colepicolo et al., 1993; Latz, Nauen, & Rohr, 2004; Seliger et al., 1969; Swift & Meunier, 1976).

We observed that the bioluminescence of *Polykrikos kofoidii* exhibits unusual flash characteristics and cellular distribution of luciferin fluorescence compared with that of other luminescent dinoflagellates. Furthermore, *P. kofoidii* appeared to contain a luciferin‐binding protein with homology to the luciferase of the heterotrophic dinoflagellate *Noctiluca scintillans*, in addition to a typical dinoflagellate luciferase with three catalytic domains, but with a luciferin‐binding‐like motif at its N‐terminal end, such as that found in a *Protoperidinium* species. These results suggested that *P. kofoidii* luciferin may be organized and bound differently from all currently described luminescent dinoflagellates.

## MATERIALS AND METHODS

### Cell culture

Cells of *Polykrikos kofoidii* were isolated from surface water samples collected from the Scripps Pier, La Jolla, California, on June 24, 2024. Cells were maintained in glass fiber filters (GF/F) and autoclaved filtered seawater (FSW) in multiwell plates at 20°C on a 12:12 h light:dark cycle of LED illumination at 50 μmol photons · m^−2^ · s^−1^. *Polykrikos kofoidii* cultures were maintained initially on a diet of *Lingulaulax polyedra* (Head et al., 2024; synonymous with *Lingulodinium polyedra*) strain CCMP 3775, a major local prey (Eppley & Harrison, 1974; Holmes et al., 1967), and then fed *Akashiwo sanguinea* strain CCMP 3776 as needed.

Other luminescent heterotrophic dinoflagellates were tested based on their occurrence in the field, being isolated as single cells from surface water samples collected at the Scripps Pier, La Jolla, California, United States. *Protoperidinium divergens* and *Pr. oblongum* cultures were established on October 27, 2023, and were maintained in sealed bottles on a rotating wheel apparatus at 20°C under room lights and fed ~1200 cells · mL^−1^ of *Lingulaulax polyedra* every 5–7 days. In addition, *Pr. steinii* cells were collected for image analysis from surface waters on November 26, 2024.

Taxonomic identity of *Polykrikos kofoidii* and *Protoperidinium divergens* was confirmed based on 18S rRNA gene and ribosomal internal transcribed spacer (ITS) rRNA region sequences. The identity of *Pr. oblongum* was based on ITS rRNA region sequence, whereas *Pr. steinii* identity was confirmed based on microscope evaluation of cell morphology during a *Pr. steinii* bloom. Cultures remained viable throughout the entire duration of the experimental phase of the project, which ended in February 2025.

Established unialgal cultures of *Ceratocorys horrida* (strain CCMP 3774 isolated by MIL from the Sargasso Sea in 1989), *Pyrocystis lunula* strain UTEX 2271, and *Py. fusiformis* (strain CCMP 3777 isolated by B. M. Sweeney in 1975 from the Halmahera Sea in Southeast Asia; Sweeney, 1981a) were grown in half‐strength Guillard's f/2‐Si medium at 20°C under cool white illumination on a 12:12 h light:dark cycle and were used for comparative measurements of bioluminescence and fluorescence emission spectra. All 18S rRNA gene and ITS rRNA region sequences are available on the GenBank database (accession numbers PX496324‐496329).

### Emission spectra measurements

Bioluminescence emission spectra were measured by an OceanOptics QE Pro spectrometer with a slit size of 25 μm, spectral resolution of 1 nm, and integration time of 1 s. Cells were suspended in 1 mL of FSW within a transparent plastic cuvette and placed in a light‐proof chamber. Light emission elicited by a 0.1‐mL injection of 1 M acetic acid was viewed by a 1‐mm UV‐transparent fiber optic light guide connected to the spectrometer. Spectra within the range of 350–700 nm were smoothed once using a Savitzky–Golay least‐square polynomial algorithm fit to a second‐degree polynomial over a 25‐channel smoothing range (Widder et al., 1983). The signal‐to‐noise ratio (SNR) for the smoothed spectrum was calculated as the ratio of the maximum intensity to the root mean square noise over the entire spectral range. The full width at half maximum (FWHM) was calculated as the spectral range at half maximum intensity. Our preliminary screening showed that placing aluminum foil as a reflective surface behind the cuvette increased SNR by at least 50%, with no significant effect on wavelength maximum or FWHM compared to the lower intensity spectra acquired without the foil. As such, all further spectra acquisitions were made using a foil for better SNR. Final spectra were based on measurements from the following cell numbers (±10% on average) per cuvette for each species: *Polykrikos kofoidii*, 10,000 cells; *Protoperidinium divergens*, 200 cells; *Ceratocorys horrida*, 3000 cells; *Pyrocystis fusiformis*, 220 cells; *Py. lunula*, 7680 cells; *Lingulaulax polyedra*, 6920 cells.

Fluorescence emission spectra and imaging were performed on individual living cells using a PARISS Hyperspectral Imaging System (LightForm Inc., Asheville, North Carolina) as previously described (Branchini et al., 2014; Holzinger et al., 2016), using a 405 ± 15 nm excitation filter with a 445‐nm long‐pass emission filter. Because single cells could be measured using this technique, we were able to measure the fluorescence emission spectra of species that had insufficient numbers for obtaining bioluminescence emission spectra.

### Mechanically stimulated flash emission measurements

Individual cells of *Polykrikos kofoidii* without visible ingested prey were isolated and suspended in 1.5 mL of filtered seawater in 7‐mL glass vials. Bioluminescence was measured within a 15‐cm diameter integrating sphere collector using a P10232 photon counting detection module with a P9111B photomultiplier having a blue‐green bialkali photocathode (ET Enterprises, Sweetwater, Texas, United States) using previous methods (Jin et al., 2013; Lindström et al., 2017). Bioluminescence was stimulated by a motorized stirrer operating at approximately 2000 rpm and measured with a 10‐ms integration duration over a 40‐s period. Photon calibration was performed with a phosphorescent tritium emitter (Letendre, Blackburn, et al., 2024), correcting for the quantum efficiency of the photomultiplier at the 450 nm phosphorescent emitter emission and the measured 475 nm dinoflagellate bioluminescence emission.

Flashes were analyzed using custom MATLAB code and verified with manual analyses. The following parameters were measured: rise time (ms), the time from initiation of a flash to maximum intensity; maximum intensity (photons · s^−1^); E‐fold time (ms), the time from maximum intensity to 1/*e* of maximum, representing initial decay rate; 90% decay time (ms), the time from maximum intensity to 10% of maximum; duration (ms), the time from flash initiation to the end of 90% decay; and integrated emission (photons · flash^−1^), the integrated emission during the flash. In addition, the total number of flashes · cell^−1^ during the 40‐s measurement period was noted.

Flash parameter values were log‐transformed prior to statistical analysis to better fit a normal distribution. Values are reported as the geometric mean of the reverse‐transformed values with 95% confidence limits based on the reverse‐transformed confidence limits of the log‐transformed values (Bland & Altman, 1996). Comparisons between the first flashes of dark versus light phase cells used an unpaired *t*‐test, whereas those of first and second flashes from the same cell used a paired *t*‐test, all with an α probability of 0.05 (*p* = 0.05).

### Confocal microscopy

Night‐phase cells of *Polykrikos kofoidii* were concentrated into 20 μL of filtered seawater by pipette. Once 20 μL of 2% low‐gelling agarose prepared in FSW had cooled, it was added to the concentrated cells (Sigma‐Aldrich, St. Louis, Missouri, United States). The 40‐μL cell suspensions were quickly transferred to slides with coverslips and sealed with nail polish for live cell imaging as noted by flagellar movement.

Live cells were imaged with a Leica Stellaris 5 Confocal Microscope at the University of California San Diego School of Medicine Microscopy Core, using a 63× (1.40 NA) oil objective. Luciferin fluorescence was visualized using 405 nm excitation and 415–495 nm emission detection band. Chlorophyll *a* fluorescence of prey cells was visualized using 485 nm excitation and 600–800 nm emission.

### Gene amplification, sequencing, and phylogenetic analysis

Approximately 50 cells of *Polykrikos kofoidii* were transferred to polymerase chain reaction (PCR) tubes containing 20 μL DNA Lysis Buffer (1% v/v Triton X‐100, 20 mM Tris–HCl pH 8, 2 mM EDTA), vortexed, and placed overnight in −20°C. Following, the PCR tubes were heated to 85°C for 10 min, diluted with 100 μL MilliQ water, and centrifuged; 1–7.5 μL were used as templates for PCR.

The PCR amplifications for the ITS rRNA region and 18S rRNA gene were performed using Takara PrimeSTAR Max DNA Polymerase ver 2 with 18ScomF‐3end and com28SR1 primer set (Wang et al., 2014; Zhang et al., 2005) and 18ScomF1 and 18ScomR1 primer set (Bai et al., 2002), respectively, with the following conditions: 98°C for 10 s, 60°C for 15 s, and 68°C for 30 s for 30 cycles (Takara, Kusatsu, Shiga, Japan). For the *lcf* gene, DinoLcfF4 and DinoLcfR1 primers were used (Valiadi et al., 2012) with the conditions: 98°C for 10 s, 52°C for 15 s, and 68°C for 30 s for 30 cycles. The PCR products were purified using a Zymo gel DNA recovery kit (Zymo, Irvine, California, United States) and Sanger sequenced at GENEWIZ (Azenta, Burlington, Massachusetts, United States).

For phylogenetic analysis, dinoflagellate luciferase (*lcf*) and luciferin binding protein (*lbp*) nucleotide and amino acid sequences obtained from GenBank were used to create a reference database (Cooney et al., 2023, 2024; Cusick et al., 2016; Gavelis et al., 2015; Johnson et al., 2019; Shih et al., 2022; Valiadi et al., 2012). Previously published bioluminescent dinoflagellate transcriptomes were downloaded as already assembled transcriptomes or as SRA files and were assembled and annotated according to Sprecher et al. (Sprecher et al., 2021; Table S1; Cooney et al., 2024, Cooney et al., 2023, Gavelis et al., 2015, Jeong et al., 2021). Benchmarking Universal Single‐Copy Orthologs (BUSCO v5.8.3) with the Alveolata (odb10) database was utilized to assess the completeness of each transcriptome (Table S1; Seppey et al., 2019). BLASTP (Altschul et al., 1990) was used to search for *lcf* or *lbp* genes in the transcriptomes with an *E*‐value of 1 × 10^−100^ and were examined manually using National Centre for Biotechology Information Basic Logal Alignment Search Tool (BLAST). Potential gene matches of *lcf* were examined to ensure the catalytic domains were present and, if possible, were split into three sections representing each of the *lcf* catalytic domains.

For all phylogenetic trees, sequences were aligned using MAFFT Alignment (Katoh et al., 2019) with default settings using Geneious version 9.1.8 and were refined manually. Maximum likelihood (ML) phylogenetic relationships for the alignments were assessed with IQ‐TREE ver. 2.1.4‐beta ModelFinder to determine the best fit model (Kalyaanamoorthy et al., 2017). Using the ideal model, 10 independent ML tree searches were conducted, and the trees with the highest log‐likelihood score were selected. Branch support was evaluated using 1000 standard nonparametric bootstrap replicates; trees were visualized in iTOL version 7.2, and *Noctiluca scintillans* was selected to re‐root the trees (Letunic & Bork, 2024).

For the luciferase nucleotide phylogenetic tree, two of the 41 unknown dinoflagellate *lcf* genes reported in Shih et al. (2022) shared high sequence similarity to *Polykrikos kofoidii* and were retained, while the remaining 39 were removed. Maximum likelihood phylogenetic relationships for the 225‐bp nucleotide alignment were created with the TIMe + G4 model.

The 281 amino acid *lcf* phylogenetic ML tree was performed using the WAG+R3 model. In addition, a Bayesian phylogenetic tree was constructed using MrBayes v3.2.6 in Geneious with the Whelan and Goldman (WAG) substitution model and an inverse gamma rate variation across four gamma categories (Huelsenbeck & Ronquist, 2001). The analysis was run for 5,000,000 generations with a burn‐in of 1,250,000 generations, four heated chains, and a subsampling frequency of 200.

For the *lbp* ML phylogenetic analysis, the 332 amino acid alignment was analyzed using the Q.pfam+G4 model. The *Polykrikos kofoidii lbp* gene sequences with homology to *Noctiluca scintillans* failed the IQ‐TREE gap‐composition test (*p* < 0.05), suggesting compositional heterogeneity, but were retained for their central relevance to the study.

AlphaFold 3 was used to generate predicted *lcf* protein structures for *Lingulaulax polyedra, Noctiluca scintillans*, and *Protoperidinium* sp.1 (Abramson et al., 2024). The *Protoperidinium* sp.1 *lcf* gene was used because of its high homology to the *P. kofoidii lcf* gene, and the *P. kofoidii lcf* gene was not full‐length. In addition, AlphaFold 3 was used to generate the *lbp* protein structure for *L. polyedra, N. scintillans lcf* gene, and *P. kofoidii lbp* gene (Abramson et al., 2024).

### Statistical analysis

Unless otherwise stated, values represent means with standard deviations. Statistical analysis was performed using JMP Pro 17 software, using analysis of variance (ANOVA) for multiple comparisons and unpaired or paired comparisons using a *t*‐test, with significance based on *p* < 0.05.

## RESULTS

### Emission spectra

**FIGURE 1:**
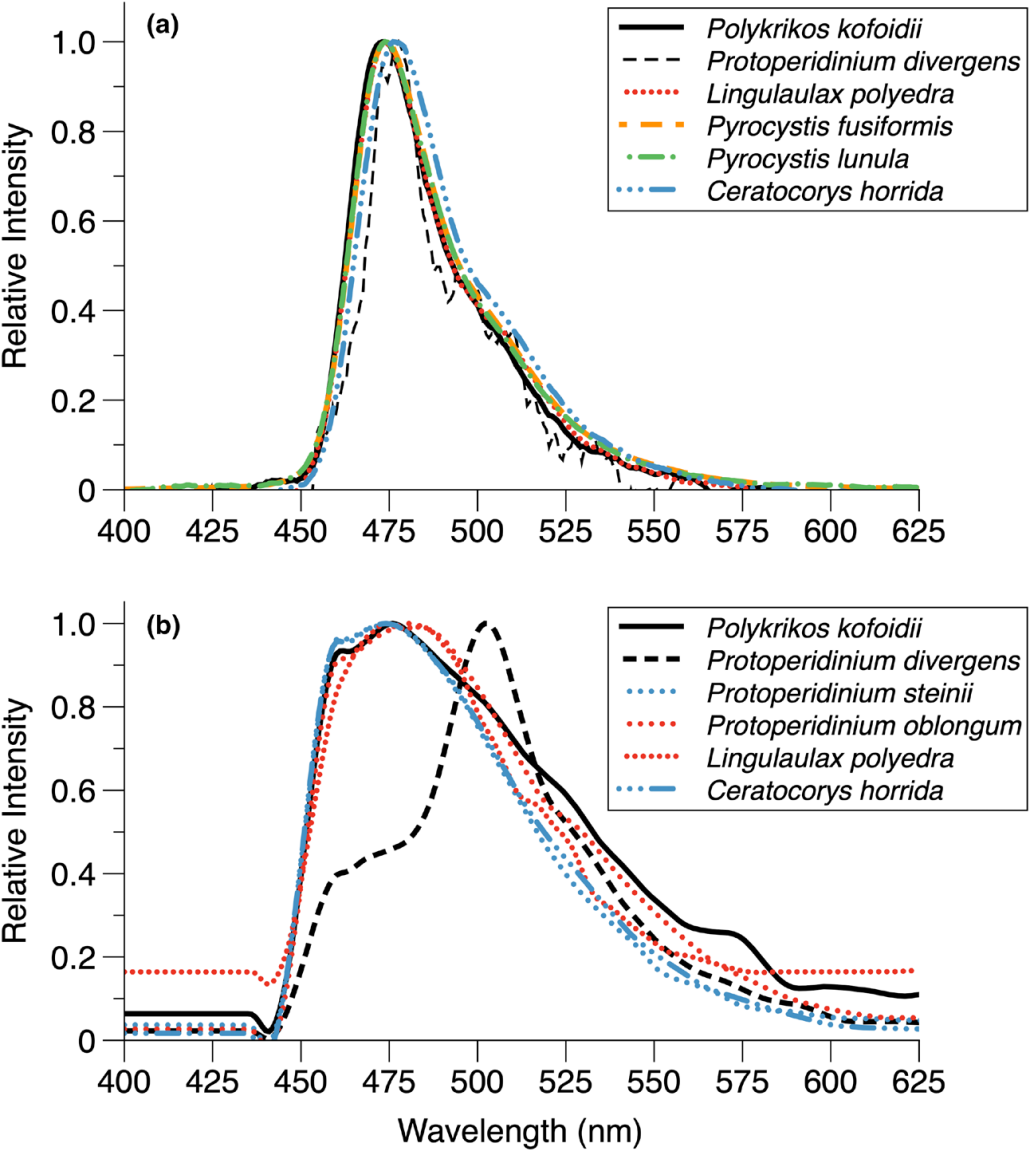
Representative emission spectra. (a) Bioluminescence emission spectra based on light emission stimulated by acetic acid treatment. (b) Fluorescence emission spectra from PARISS hyperspectral imaging, not including chlorophyll fluorescence from the autotrophic species. Each spectrum has been smoothed once.

The bioluminescence emission spectrum of *Polykrikos kofoidii* had a maximum emission at 473.5 nm, with a FWHM of 28.7 nm (Figure 1a), similar to those of other dinoflagellates (Table 1). For all dinoflagellates measured, the maximum emission was at 474.7 ± 1.6 nm, with a FWHM of 30.0 ± 3.4 nm (*n* = 25), indicating a similar bioluminescent chemistry.

**TABLE 1:** Bioluminescence emission spectra measured during chemical stimulation by acetic acid, with spectra smoothed once.

Species	Maximum wavelength (nm)	FWHM (nm)	SNR (range)
*Polykrikos kofoidii*	473.5 ± 0.5 (3)	28.7 ± 0.8	12–23
*Protoperidinium divergens*	476.9 ± 1.1 (2)	19.8 ± 1.7	2–3
*Pyrocystis fusiformis*	473.7 ± 0.6 (6)	30.4 ± 1.1	36–578
*Pyrocystis lunula*	474.0 ± 0.4 (3)	30.6 ± 0.5	41–84
*Lingulaulax polyedra*	473.3 ± 0.5 (3)	30.3 ± 0.8	26–29
*Ceratocorys horrida*	476.2 ± 0.7 (8)	32.4 ± 0.7	26–48

*Note*: FWHM is the full width of the spectrum at half maximum intensity, and SNR is the signal‐to‐noise ratio of the smoothed spectrum after subtracting the background. Values represent means with standard deviations, with the number of independent samples measured in parentheses.

The fluorescence emission spectrum of *Polykrikos kofoidii* had a maximum emission at 477.3 ± 2.3 nm, with a FWHM of 86.0 ± 07.9 nm (Figure 1b, Table 2). *Protoperidinium steinii*, *Pr. oblongum*, *Lingulaulax polyedra*, and *Ceratocorys horrida* had similar fluorescence emission spectra (ANOVA: *F*_4,14_ = 0.5, *p* = 0.9), comparable to the fluorescence emission peak of 474 nm for *Pyrocystis* luciferin (Dunlap & Hastings, 1981a). However, *Pr. divergens* exhibited bright fluorescence with a peak at 504.3 ± 2.1 nm with a much narrower FWHM of 33.7 ± 9.0 nm (Table 2), indicating that another fluorescent compound was present. At low SNR, a shoulder on the shorter wavelength side of the spectrum was evident (Figure 1b), corresponding to the wavelength peak found for the other dinoflagellates.

**TABLE 2:** Fluorescence emission spectra measured using the PARISS hyperspectral imaging system, with spectra smoothed once.

Species	Maximum wavelength (nm)	FWHM (nm)	SNR (range)
*Polykrikos kofoidii*	477.3 ± 2.3 (3)	86.0 ± 7.9	13–16
*Protoperidinium steinii*	475.0 ± 1.0 (3)	68.0 ± 3.6	19–27
*Protoperidinium oblongum*	476.5 ± 6.4 (2)	71.5 ± 7.8	30–37
*Ceratocorys horrida*	477.0 ± 3.6 (4)	68.8 ± 2.4	11–59
*Lingulaulax polyedra*	476.3 ± 2.1 (3)	73.7 ± 5.5	4–7
*Protoperidinium divergens*	504.3 ± 2.1 (3)	33.7 ± 9.0	44–162

*Note*: FWHM is the full width of the spectrum at half maximum intensity, and SNR is the signal‐to‐noise ratio of the smoothed spectrum after subtracting the background. Values represent means with standard deviations, with the number of independent measurements in parentheses. Chlorophyll fluorescence spectra are not included.

The autotrophic dinoflagellates *Ceratocorys horrida*, *Pyrocystis fusiformis*, and *Py. lunula* displayed similar chlorophyll fluorescence emission spectra, with a wavelength maximum at 670.1 ± 1.7 nm, with a FWHM of 26.1 ± 1.4 nm (*n* = 12).

### Bioluminescence flash kinetics characteristics

#### *Polykrikos kofoidii* flashes

**FIGURE 2:**
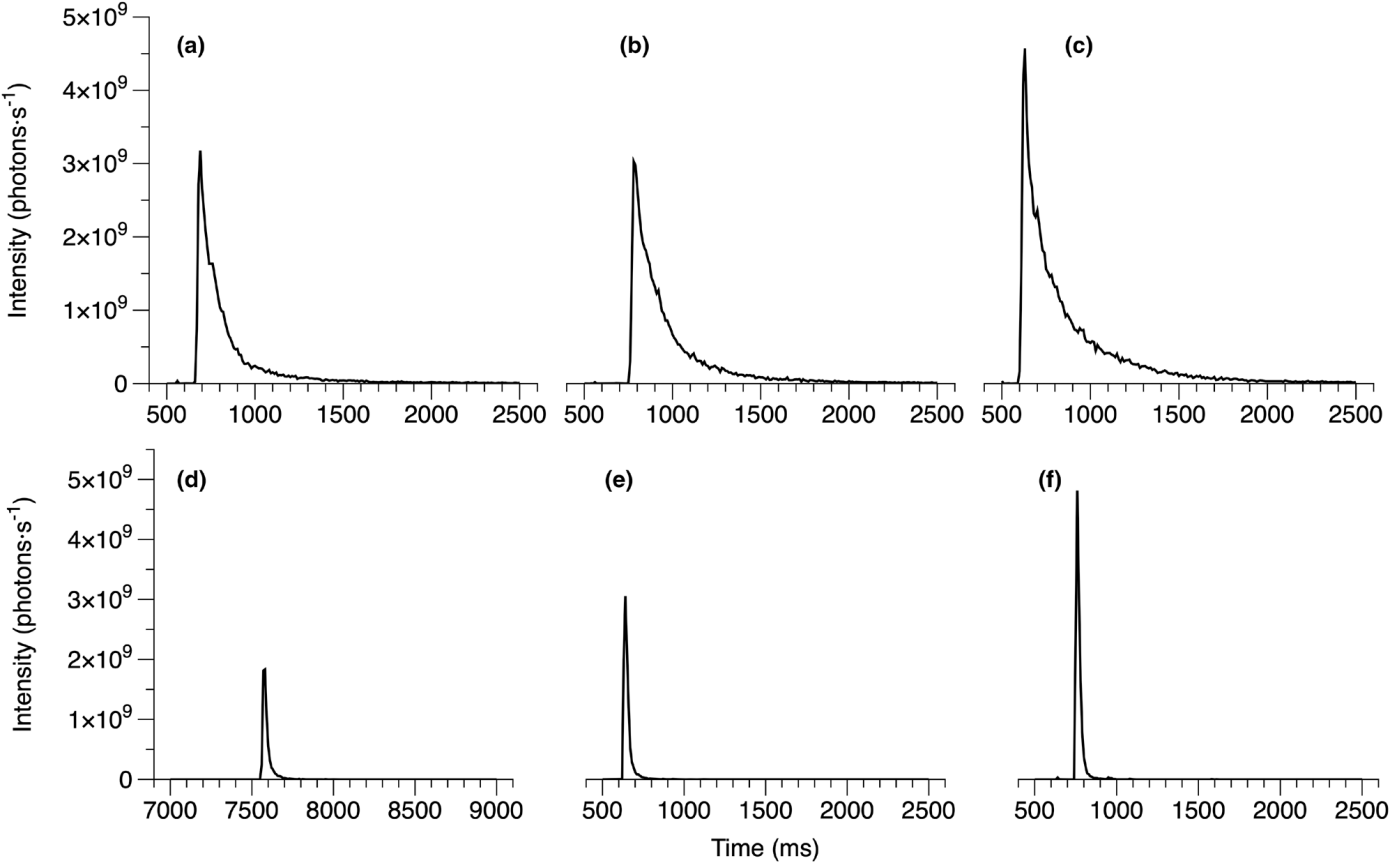
Representative records of the first flashes of individual (a–c) *Polykrikos kofoidii* and (d–f) *Protoperidinium divergens* dark phase cells.

*Polykrikos kofoidii* dark phase cells produced 3.8 ± 2.6 (*n* = 34) flashes · cell^−1^ within the 40‐s measurement period. The first flashes of each cell had an average rise time of 49 ms, E‐fold time of 108 ms, 90% decay time of 275 ms, and total duration of 327 ms, with a maximum flash intensity of 3.9 × 10^8^ photons · s^−1^ and integrated emission of 5.5 × 10^9^ photons · flash^−1^ (Figure 2, Table 3). Second flashes were longer with lower photon emission (Table 3).

**TABLE 3:** Bioluminescence flash kinetics characteristics for dark phase cells of *Polykrikos kofoidii and Protoperidinium divergens*.

Condition	Rise time (ms)	Maximum intensity (photons · s^−1^)	E‐fold time (ms)	90% decay time (ms)	Total duration (ms)	Total emission (photons)
*Polykrikos kofoidii* (26)						
First flash	49.0* (44–55)	3.87 × 10^8^* (2.28–6.58 × 10^8^)	107.7 (85–136)	275.0* (247–306)	326.7 (299–357)	5.51 × 10^9^* (3.03–10.1 × 10^9^)
Second flash	60.2* (50–73)	1.03 × 10^8^* (0.74–1.44 × 10^8^)	133.8 (105–171)	380.6* (294–492)	411.2 (323–524)	1.47 × 10^9^* (0.83–2.60 × 10^9^)
*Protoperidinium divergens* (16)						
First flash	24.1 (20.3–28.7)	9.46 × 10^8^ (6.04–14.8 × 10^8^)	43.3 (33.3–56.3)	87.0 (66.2–114.1)	115.0 (92.3–143.3)	4.64 × 10^9^* (2.80–7.67 × 10^9^)
Second flash	23.1 (20.4–26.1)	5.23 × 10^8^ (3.08–8.86 × 10^8^)	34.0 (28.7–40.2)	70.5 (57.9–85.8)	95.1 (81.3–111.3)	2.00 × 10^9^* (1.17–3.41 × 10^9^)

*Note*: Values represent geometric means with 95% confidence limits in parentheses. The number of cells measured is indicated in parentheses in the Condition column. An asterisk (*) denotes a significant difference in the parameter between the first and second flashes of each cell within the species, based on a *t*‐test.

Each cell produced 3.6 ± 2.7 (*n* = 37) flashes during the light phase, a value not significantly different from that produced by dark phase cells (*t*‐test, *t* = 0.32, *df* = 69, *p* = 0.75). Light phase flashes had faster kinetics and reduced photon emission that were significantly different from that of dark phase flashes (Table 4).

**TABLE 4:** Characteristics of the first flashes of *Polykrikos kofoidii* based on measurements during the light and dark phases of the daily cycle.

Condition	Rise time (ms)	Maximum intensity (photons · s^−1^)	E‐fold time (ms)	90% decay time (ms)	Total duration (ms)	Total emission (photons)
Light	41.6* (37.0–46.6)	1.10 × 10^8^* (0.78–1.55 × 10^8^)	87.7 (74.6–103.0)	208.2* (182.7–237.4)	253.8* (226.6–284.2)	1.16 × 10^9^* (0.75–1.81 × 10^9^)
Dark	50.0* (45.4–54.9)	3.95 × 10^8^* (2.34–6.64 × 10^8^)	110.0 (61.1–132.8)	288.9* (262.3–318.2)	341.5* (314.3–371.0)	5.78 × 10^9^* (2.29–10.2 × 10^9^)

*Note*: Values represent geometric means with 95% confidence limits in parentheses for 33 cells. Asterisk (*) represents a significant difference in the parameter between light and dark first flashes, based on a *t*‐test.

#### *Protoperidinium divergens* flashes

The flashes of *Protoperidinium divergens* dark phase cells were measured for comparison. Each cell produced 2.5 ± 2.4 (*n* = 27) flashes · cell^−1^ within the measurement period. The first flashes of each cell had a rise time of 24 ms, E‐fold time of 43 ms, 90% decay time of 87 ms, and total duration of 115 ms, with a maximum intensity of 9.5 × 10^8^ photons · s^−1^ and integrated emission of 4.6 × 10^9^ photons · flash^−1^ (Figure 2, Table 3). Second flashes had slightly faster kinetics and lower maximum intensity, although only the decrease in integrated flash emission was significantly different (Table 3).

### Confocal microscopy

**FIGURE 3:**
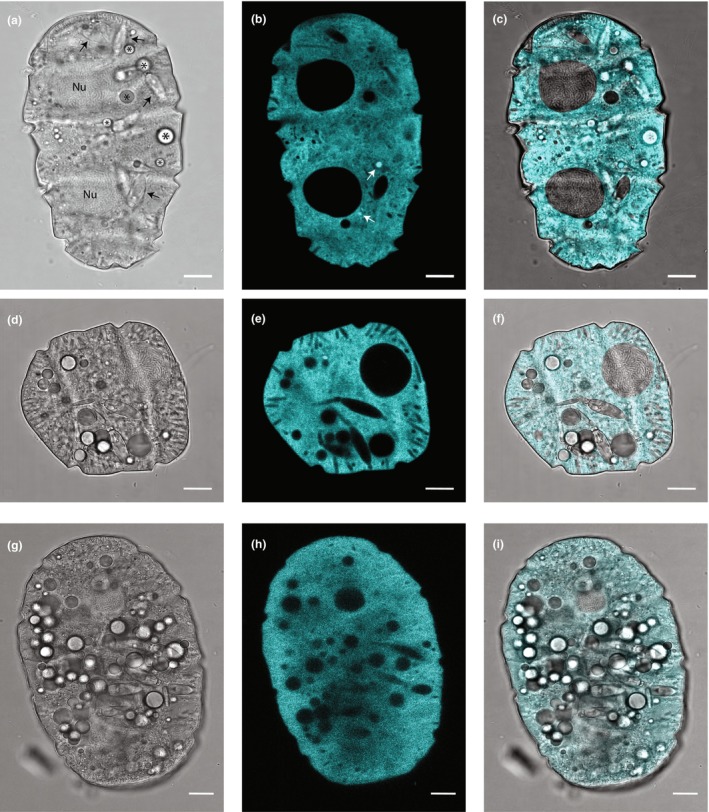
Laser confocal microscope images of three living *Polykrikos kofoidii* dark phase cells. Left column (a, d, g): Transmitted light images. Center column (b, e, h): Diffuse fluorescence. Right column (c, f, i): Merged transmitted and fluorescence images. The nuclei (‘Nu’ in a) are visible as large dark objects, along with nematocysts (shown with black arrows in a), lipid droplets (* in a), and vesicles with brighter fluorescence (shown with white arrows in b). Scale bars represent 10 μm.

**FIGURE 4:**
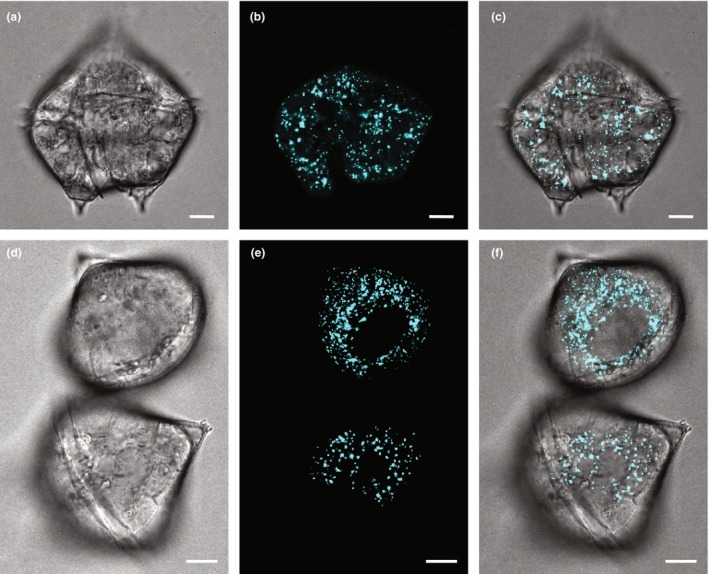
Laser confocal microscope images of three living *Protoperidinium divergens* dark phase cells. Left column (a, d): Transmitted light images. Center column (b, e): Punctate fluorescence from luciferin. Right column (c, f): Merged transmitted and fluorescence images. Scale bars represent 10 μm.

*Polykrikos kofoidii* cells contained four sulcus and two nuclei, with young cells containing two sulcus and one nucleus (Figure 3). Fluorescence excited by 405 nm excitation was diffuse throughout the cell (Figure 3, S1), with occasionally only a few vesicles showing brighter fluorescence (Figure 3b). Also evident was a paracrystalline pattern to the condensed chromosomes within the nucleus, numerous nematocysts, which were occasionally observed discharged into the surrounding fluid, and lipid droplets. In contrast, cells of the other heterotrophic species measured clearly exhibited punctate sources of fluorescence due to 405 nm excitation, consistent with luciferin fluorescence originating from scintillons and no diffuse fluorescence (Figure 4, S1).

**FIGURE 5:**
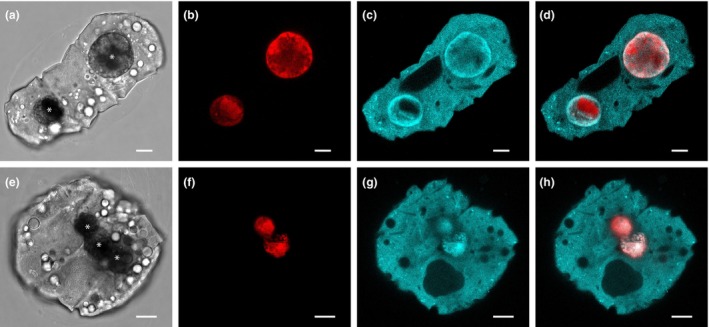
Laser confocal microscope images of two living *Polykrikos kofoidii* dark phase cells containing intact *Lingulaulax polyedra* prey (*). The top *P. kofoidii* cell contains two *L. polyedra* prey cells, while the bottom *P. kofoidii* cell contains three prey cells. Left column (a, e): Transmitted light images. Center left column (b, f): *L. polyedra* chlorophyll fluorescence. Center right column (c, g): Diffuse *P. kofoidii* fluorescence and *L. polyedra* fluorescence. Right column (d, h): Merged transmitted and fluorescence images. Scale bars represent 10 μm.

Some cells of *Polykrikos kofoidii*, which is a raptorial feeder, contained intact prey cells, in this case *Lingulaulax polyedra*. Engulfed prey cells exhibited blue‐green luciferin fluorescence with 405 nm excitation and bright red chlorophyll fluorescence with 485 nm excitation (Figure 5).

### Analysis of bioluminescence genes

All deposited *lcf* and *lbp* gene sequences were downloaded from GenBank, and 20 previously published bioluminescent dinoflagellate transcriptomes were used to search for *lcf* and *lbp* genes (Table S1). No *lcf* or *lbp* genes were detected in the *Polykrikos lebouriae, Protoperidinium* sp.2, or *Noctiluca scintillans* transcriptomes.

#### Luciferase nucleotide phylogeny

**FIGURE 6:**
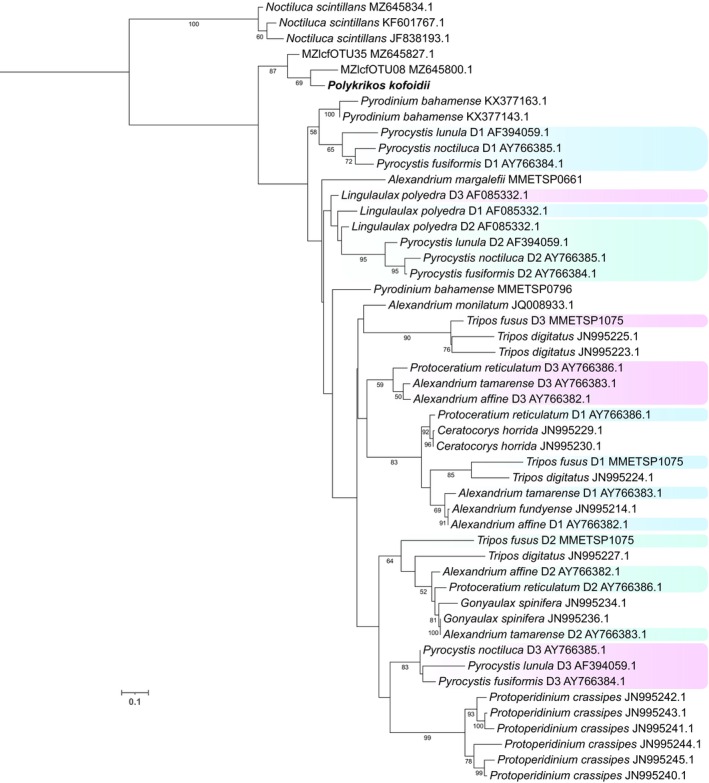
Maximum likelihood phylogenetic tree based on the alignment of dinoflagellate luciferase catalytic domain nucleotide sequences. Branch support using 1000 standard nonparametric bootstrap replicates is shown. When multiple catalytic domains were present, each was included and labeled D1 (highlighted in blue), D2 (highlighted in green), or D3 (highlighted in purple), followed by GenBank accession numbers. *Polykrikos kofoidii* sequence is labeled in bold.

An ML phylogenetic tree of dinoflagellate *lcf* catalytic domain nucleotide sequences showed species‐level clustering across the three dinoflagellate luciferase domains with moderate to high bootstrap support (Figure 6). Sequences from closely related species were consistently grouped together, indicating strong evolutionary conservation. Moreover, the three luciferase domains (D1, D2, and D3) generally formed distinct, non‐overlapping clades, with the primary exception being *Lingulaulax polyedra*, in which the three different domains clustered together. *Polykrikos kofoidii*, alongside two unidentified dinoflagellate sequences, formed a separate branch from the other luminescent dinoflagellates.

#### Luciferase amino acid phylogeny

**FIGURE 7:**
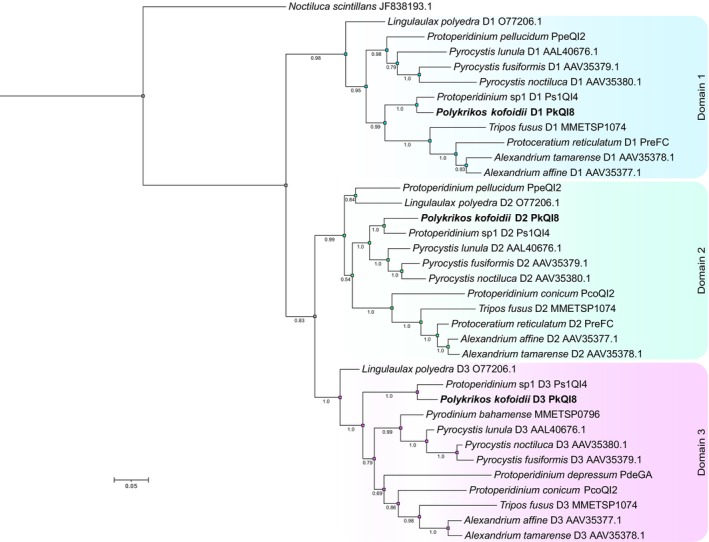
Maximum likelihood phylogenetic tree based on the alignment of dinoflagellate luciferase catalytic domain amino acid sequences, showing 1000 nonparametric bootstrap values. Luciferase catalytic domain 1 is highlighted in blue (D1), domain 2 in green (D2), and domain 3 in purple (D3). GenBank accession numbers or transcriptome names are shown at the end of the label. *Polykrikos kofoidii* sequences are labeled in bold.

An ML phylogenetic analysis of dinoflagellate luciferase amino acid sequences revealed strong domain‐level separation (Figure 7). Sequences corresponding to domains D1, D2, and D3 formed distinct, non‐overlapping clades for all luminescent dinoflagellates. The D2 and D3 were more closely related to each other than to D1, and each domain had a distinct internal topology. *Polykrikos kofoidii* consistently grouped with a *Protoperidinium* species, forming a well‐supported clade. Overall, genera tended to cluster together within each domain, although *Protoperidinium* species were more dispersed and did not consistently form cohesive clades. Bootstrap values at major nodes were consistently high, and these relationships were further supported by a Bayesian phylogenetic analysis (Figure S2).

#### Luciferin‐binding protein amino acid phylogeny

**FIGURE 8:**
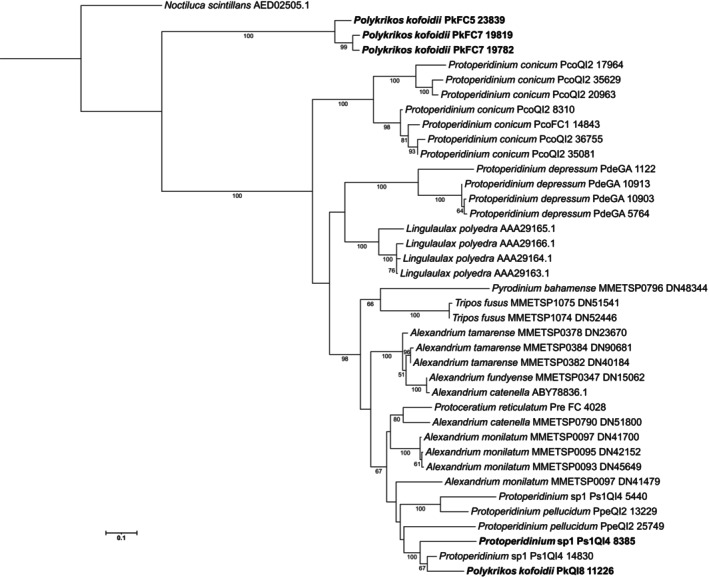
Luciferin binding protein maximum likelihood phylogenetic tree showing 1000 nonparametric bootstrap values. GenBank accession numbers or transcriptome names are shown at the end of each dinoflagellate species label. *Polykrikos kofoidii* PkFC5/7 *lbp* sequences are labeled in bold along with the *lcf* N‐terminal *lbp*‐like motif domain for *Protoperidinium* sp.1 and *P. kofoidii* PkQ18.

An ML phylogenetic analysis of dinoflagellate luciferin binding protein sequences revealed *Polykrikos kofoidii* and *Protoperidinium* sp.1 *lcf* N‐terminal domains grouped together and along with other *Protoperidinium pellucidum* and *Alexandrium* species (Figure 8). *Polykrikos kofoidii lbp* was identified in three of the nine available *P. kofoidii* transcriptomes and was distinct from other known *lbp* gene sequences, sharing similar but not exact homology to *Noctiluca scintillans* hybrid *lcf‐lbp* protein. These *P. kofoidii lbp* gene sequences consistently grouped together, away from the other bioluminescent dinoflagellates and near *N. scintillans* (Figure 8). Overall, species tended to group together and form separate clades. *Alexandrium* species tended to form strongly supported monophyletic groups, whereas *Protoperidinium* sequences were dispersed across the trees.

### Protein structures

**FIGURE 9:**
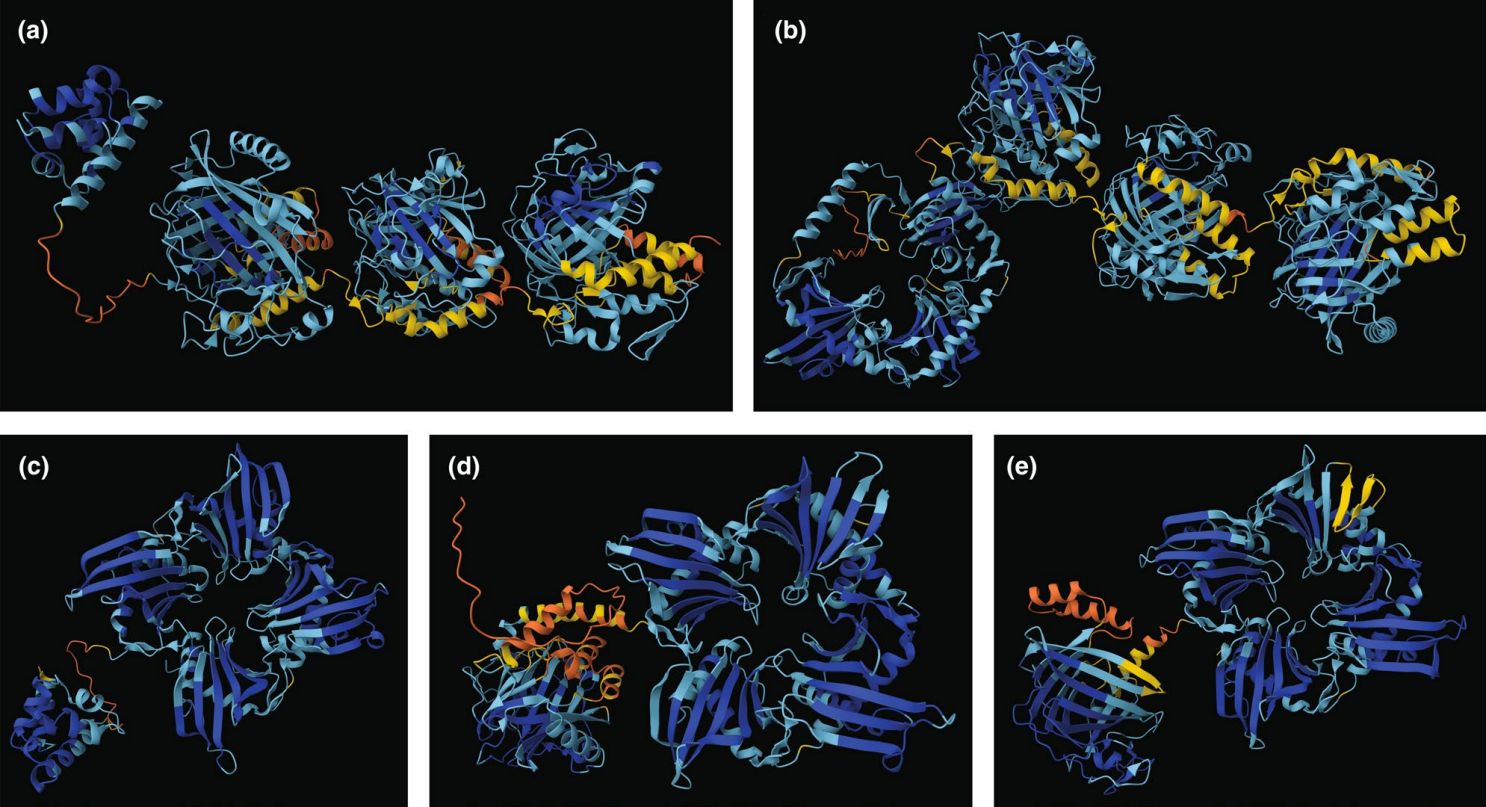
Alphafold 3 predicted structure for luciferase, LCF, and luciferin binding proteins, LBP. Colors correspond to per‐residue structure confidences (pLDDT): Dark blue, pLDDT > 90; light blue, 90 > pLDDT > 70, yellow, 70 > pLDDT > 50, and orange, pLDDT < 50. (a) *Lingulaulax polyedra* LCF; (b) *Protoperidinium* sp.1 LCF; (c) *L. polyedra* LBP; (d) *Noctiluca scintillans* LCF; (e) *Polykrikos kofoidii* LBP.

Alphafold 3 was used to generate 3D protein structures of *Lingulaulax polyedra lcf* and *lbp, Noctiluca scintillans lcf*, *Protoperidinium* sp.1 *lcf*, and *Polykrikos kofoidii lbp* (Figure 9). The N‐terminal domain of the *Protoperidinium* sp.1 *lcf* gene was larger (Figure 9b), with a structure similar to that of *Lingulaulax polyedra* (Figure 9c) and *Noctiluca scintillans lbp* domains (Figure 9d), which was also evident on the amino acid level and in the *lbp* phylogenetic tree (Figure 8, Figure S3). The three catalytic domains of *lcf* were very similar in structure for both *L. polyedra* (Figure 9a) and *Protoperidinium* sp.1 (Figure 9b).

## DISCUSSION

The bioluminescence of *Polykrikos kofoidii* exhibited characteristics that distinguished it from that of other dinoflagellates, with slow dimmer flashes and diffuse fluorescence consistent with the spectral emission of luciferin distributed throughout the cell. It produced flashes during the light phase, but with faster kinetics and reduced photon emission when compared to its dark phase flashes.

The bioluminescence emission spectrum of *Polykrikos kofoidii*, with a peak at 474 nm, was similar to that of other dinoflagellates (Letendre, Twardowski, et al., 2024), which all involve the same luminescent chemistry (Schmitter et al., 1976; Valiadi & Iglesias‐Rodriguez, 2013), suggesting converging evolution for this property across these different taxa.

### Unique aspects of autofluorescence

Bioluminescence in dinoflagellates originates from discrete organelles known as scintillons, vesicles that contain the luminescent chemistry (Desa & Hastings, 1968; Hastings & Dunlap, 1986; Nicolas et al., 1991) and exhibit fluorescence with maximum emission at 475 nm upon UV excitation (Johnson et al., 1985) due to the autofluorescence of luciferin (Dunlap & Hastings, 1981a; Hastings & Dunlap, 1986; Nakamura et al., 1989). The fluorescence emission maximum at 477 nm for *Polykrikos kofoidii* is consistent with the presence of luciferin, indicating that the observed diffuse fluorescence at 405 nm excitation was due to luciferin. *Protoperidinium steinii* and *Pr. oblongum* also exhibited similar fluorescence emission spectra, consistent with the fluorescence of luciferin.

The observed autofluorescence in *Polykrikos kofoidii* was distributed diffusely across the cytoplasm with occasional small punctate sources with brighter fluorescence, unlike the typical pattern observed here in *Protoperidinium divergens* and in all other studied dinoflagellates, in which luciferin fluorescence has originated solely from the scintillons as punctate sources (Dagenais‐Bellefeuille et al., 2017; Eckert & Reynolds, 1967; Fritz et al., 1990; Johnson et al., 1985; Latz & Lee, 1995; Seo & Fritz, 2000; Sweeney, 1978; Widder & Case, 1981b). As hyperspectral imaging revealed only one fluorescence emission spectrum similar to that of luciferin, the observed diffuse fluorescence in *P. kofoidii* is presumed to originate from luciferin.

In *Protoperidinium divergens*, the autofluorescence of luciferin was eclipsed by a second much brighter fluorescence with a peak emission at 504 nm. This emission was similar to that measured for *Pr*. cf. *brevipes*, which had a peak at 495 nm (Carpenter et al., 1991), but different from so‐called green‐fluorescent dinoflagellates, diatoms, and other microalgae, which have peak fluorescent emissions of ~530–540 nm (Shapiro et al., 1989; Tang & Dobbs, 2007). The bright fluorescence from *Pr. divergens* is indicative of a different fluorescent compound that has not yet been described.

### Speculation on the diffuse distribution of *Polykrikos kofoidii* luciferin

The widespread distribution of luciferin outside of scintillons within the *Polykrikos kofoidii* cell is quite unusual and needs further study. Luciferin is protected from autooxidation by binding to LBP at physiological pH (Fajardo et al., 2020; Morse, Pappenheimer, et al., 1989; Valiadi & Iglesias‐Rodriguez, 2014), and oxidized luciferin is not fluorescent (Dunlap & Hastings, 1981a; Johnson et al., 1985). So, if luciferin is distributed throughout the cell, based on its fluorescence mapping, then so would be LBP. LBP is a large and diverse gene family (Lee et al., 1993; Valiadi & Iglesias‐Rodriguez, 2014) that is one of the most highly expressed proteins in dinoflagellates (Tanikawa et al., 2004), dominating the transcript pool (Erdner & Anderson, 2006; Toulza et al., 2010; Uribe et al., 2008) and accounting for about 1% of the total proteome (Morse, Pappenheimer, et al., 1989). LBP is a highly abundant protein, about 100 times more abundant than luciferin (Dunlap & Hastings, 1981b), and is not expected to be limited in its abundance within the cell. It is also not fluorescent.

Dinoflagellate bioluminescence is very similar across taxa and involves activation of the luminescent chemistry within scintillons that are closely associated with the vacuole membrane, where the opening of voltage‐gated proton channels (Rodriguez et al., 2017; Smith et al., 2011) following the release of Ca^2+^ from intracellular stores (von Dassow & Latz, 2002) results in a decrease in pH within the scintillon, causing dissociation of luciferin from LBP, its subsequent binding to luciferase, and oxidation leading to light emission (Fogel & Hastings, 1971, 1972; Nawata & Sibaoka, 1979; Schultz et al., 2005). We speculate that the luciferin that is widely distributed is not associated with the vacuole membrane and not involved in the luminescent flash but, rather, serves as a storage pool. Luciferin, luciferase, and LBP are packaged into scintillons (Desjardins & Morse, 1993; Nicolas et al., 1991; Schmitter et al., 1976) and translocated within the cell to the periphery near the vacuole membrane. The mechanisms by which luciferin and LBP associate independently of scintillons remain to be determined.

Future work can investigate the spatial distribution of bioluminescence emission within the *Polykrikos kofoidii* cell, determine the colocalization of LBP relative to that of luciferin, compare pre‐ to post‐bioluminescence luciferin fluorescence to determine the sites of light emission, and determine whether there are day‐night differences in luciferin abundance, which occur in *Lingulaulax polyedra* (Fritz et al., 1990) but not in *Pyrocystis lunula* (Colepicolo et al., 1993; Knaust et al., 1998).

### Inferences based on the phylogeny of dinoflagellate bioluminescence genes

Comparison of the nucleotide and amino acid luciferase phylogenetic trees revealed notable differences in the inferred evolutionary relationships among luciferase sequences. Both trees supported strong species‐level clustering and domain‐level separation, yet the amino acid tree more clearly resolved the divergence among the three catalytic domains (D1, D2, D3), with domain‐specific clades forming consistently across taxa, as previously documented (Liu et al., 2004). In contrast, the nucleotide tree had the multiple domains intertwined throughout the tree, and for *Lingulaulax, polyedra* the domains grouped together, suggesting lower domain sequence divergence at the nucleotide level, potentially due to synonymous substitutions. *Protoperidinium* species consistently did not form genus‐level clusters, suggesting luciferase acquisition may have occurred multiple times in this genus or they have significantly different mutation rates.

Only one or two of the three catalytic domains were observed in *Pyrodinium bahamense, Protoperidinium conicum, Pr. depressum*, and *Pr. pellucidum* transcriptomes. The *lcf* protein is very large (~137 kDa), making it difficult to capture the full‐length gene. Furthermore, all three *Protoperidinium* species had only 32%–41% Busco completeness; thus, it is unknown at this time if these species possess all three catalytic domains but were just not fully recovered in the currently available transcriptomic datasets.

The nine publicly available *Polykrikos kofoidii* transcriptomes used in this study were generated from a starved laboratory‐grown culture fed *Alexandrium minutum* and from eight single cells isolated from waters off British Columbia, Canada (Cooney et al., 2023; Jeong et al., 2021). The *lcf* amino acid sequence reported here was selected as it was the most complete in length, because multiple contigs from all transcriptomes overlapped with this transcript, and because the translated PCR amplified *lcf* nucleotide sequence reported here matched to its *lcf* domain 1 sequence. However, the sequence was not full‐length, as it did not begin with a methionine. The full length of the protein remains uncertain, but if it is comparable to the *Protoperidinium* sp. 1 sequence, it would be approximately 177 amino acids longer.

The N‐terminal domain of *lcf* is variable among bioluminescent dinoflagellates, and its exact function is unknown (Fajardo et al., 2020). Interestingly, based on amino acid alignment and predicted protein structure, *Polykrikos kofoidii* and *Protoperidinium* sp.1 appear to have an *lbp*‐type motif on the N‐terminal domain of their *lcf*. In both *Noctiluca scintillans* and *Lingulaulax polyedra*, the LBP has four internal repeat domains (Liu & Hastings, 2007). The *lbp* motif observed in this study did not contain all four internal domains; therefore, it remains unknown if the *P. kofoidii* and *Protoperidinium* sp.1 N‐terminal *lbp*‐like motifs act to bind and protect luciferin from autooxidation or if they serves another, unrelated function.

Finding the *lbp* with close homology to *Noctiluca scintillans lcf‐lbp* hybrid protein in three of the nine available *Polykrikos kofoidii* transcriptome is noteworthy, but it is peculiar that it was not recovered in all transcriptomes. LBP abundance was shown as higher during the night phase in *Lingulaulax polyedra*, yet the *lbp* mRNA transcript levels did not vary across day or night (Morse, Milos, et al., 1989). If *P. kofoidii* transcriptomes were harvested during the day phase and their mRNA transcripts vary over the daily cycle, then perhaps there were few transcripts available to sequence in the other transcriptomes, or it is possible this gene is strain‐specific or potentially from an unknown bioluminescent prey transcript inside *P. kofoidii*.

Generating transcriptomes for heterotrophic species is challenging, as prey contamination is always a concern and must be carefully considered during sequence analysis. This issue is further complicated for bioluminescence‐related genes, as prey contaminated transcripts would also yield a dinoflagellate genetic signal. The LBP sequence was detected exclusively in single‐cell‐generated transcriptomes. The cells used to generate the transcriptomes with the two longest transcripts reported here (PkFC5 and PkFC7) lacked apparent prey in their cells and were sampled at different time points (Figure S1E,G, Table S1; Cooney et al., 2023). Across the three single‐cell transcriptomes containing this gene, 14 transcripts overlapped with the reported proteins at greater than 90% identity, and when combined, the reconstructed protein is expected to be nearly full‐length, reaching a comparable length to *Noctiluca scintillans* protein. However, to fully rule out prey contamination and to determine the entire length of the gene, amplification from gDNA is necessary.

### Implications of the unique flash characteristics of *Polykrikos kofoidii*

A commonly used parameter of dinoflagellate bioluminescence is Total Mechanically Stimulable Bioluminescence (TMSL), an index of mechanically stimulated bioluminescence capacity per cell (Batchelder & Swift, 1989; Biggley et al., 1969; Buskey et al., 1992; Lapota, Young, et al., 1992; Park et al., 2024b; Seliger et al., 1969; Sweeney, 1981b; Swift et al., 1995). However, more relevant in identifying planktonic sources of bioluminescence measured by bathyphotometers is to discriminate flashes based on their bioluminescence signature, mainly flash intensity and kinetics (Cronin et al., 2016; Johnsen et al., 2014; Letendre, Blackburn, et al., 2024; Messié et al., 2019; Moline et al., 2009). Flash kinetics measured by bathyphotometers may be different from those from laboratory measurements within an integrating light collector because as the organisms are advected through the instrument, the flow path, recirculation, and detector configuration may alter the position of flashing organisms relative to the detector (Herren et al., 2005; Letendre, Blackburn, et al., 2024; Orrico et al., 2009). For example, the 40‐ms rise time of *Lingulaulax polyedra* flashes measured in the UBAT bathyphotometer (Letendre, Blackburn, et al., 2024) was longer than the 22–34‐ms rise time measured in a laboratory integrating light collector (Latz & Lee, 1995). Nonetheless, bioluminescence signatures show that dinoflagellates can be discriminated from other luminescent sources such as crustaceans and gelatinous zooplankton based on their fast flash kinetics and dimmer intensity (Cronin et al., 2016; Johnsen et al., 2014; Letendre, Blackburn, et al., 2024; Messié et al., 2019).

In the present study, the flashes of *Polykrikos kofoidii* had much slower kinetics than those of *Protoperidinium divergens*, with a rise time twice as long and a flash duration approximately three times greater. Considering that flashes of *Protoperidinium* sp. measured with the UBAT bathyphotometer had a rise time of 50–80 ms and duration of 350 ms (Cronin et al., 2016; Xue et al., 2020), we predict that *P. kofoidii* flashes similarly measured would exhibit very long flash kinetics. Once *P. kofoidii* flash characteristics are included in libraries of flash signatures, then the success of classification schemes based on bathyphotometer flash analyses can be evaluated.

Diel changes in field‐measured bioluminescence (Marcinko et al., 2013) are due in part to a reduction in dinoflagellate bioluminescence potential (Hamman & Seliger, 1972) and flash intensity during the day, which occur in many dinoflagellates including heterotrophic species (Buskey et al., 1994; Lapota, Young, et al., 1992). In the present study, cells of *Polykrikos kofoidii* produced a similar number of flashes during the light phase as during the dark phase, although day‐phase flashes had faster kinetics and reduced intensities relative to night‐phase flashes. These results indicate that the bioluminescence system of *P. kofoidii* still functions during the day phase, although with dimmer flashes.

Diel differences in dinoflagellate bioluminescence can be due to a circadian rhythm in availability of luciferin, luciferase, or luciferin‐binding protein; a reduction in the number of scintillons during the day or translocation of scintillons during the day so that they are not excitable; photoinhibition, a decrease in bioluminescence due to light exposure during the night phase; or a combination of these factors (Buskey et al., 1992; Fritz et al., 1990; Marcinko et al., 2013; Seo & Fritz, 2000; Valiadi & Iglesias‐Rodriguez, 2013). Dark phase photoinhibition of *Polykrikos kofoidii* was not investigated in this study, but the bioluminescence of the related *P. swartzii* was reported not to be inhibited by light exposure during the dark phase (Hamman & Seliger, 1972). The diel changes in *P. kofoidii* flash kinetics observed in this study, namely reduced flash intensity and faster kinetics during the day phase, suggest that its bioluminescence may be photoinhibited during the dark phase.

### *Polykrikos kofoidii* as a dinoflagellate predator and its impact on measurements of primary production in the oceans

A major challenge in identifying functional groups in marine ecosystems is to distinguish between primary and secondary consumers. Bioluminescence flash intensity and kinetics are useful parameters for distinguishing source identity, especially between dinoflagellates and zooplankton (Berge et al., 2012; Cronin et al., 2016; Moline et al., 2009). Dinoflagellates, as major grazers as well as important prey, are members of both the autotrophic and heterotrophic plankton communities. Fluorescence microscopy has been used to distinguish between chlorophyll‐containing and heterotrophic dinoflagellates (Lessard & Swift, 1986). Although in situ measurements of chlorophyll fluorescence sample mostly autotrophs, bioluminescence measurements are effective in sampling both autotrophic and heterotrophic luminescent dinoflagellates, which can be distinguished on the basis of flash emission properties (Letendre, Blackburn, et al., 2024). Measurements of bioluminescence, in tandem with other parameters such as chlorophyll fluorescence, can provide insights into the composition of plankton communities.

For grazers such as *Polykrikos kofoidii* that ingest whole prey that retain their chlorophyll fluorescence, measurements of in situ chlorophyll fluorescence—for example, using fluorometry, the imaging flow cytobot (Olson & Sosik, 2007), and remote sensing (Cetinic et al., 2024; Cullen et al., 1997)—may overestimate the abundance of autotrophs if they detect chlorophyll fluorescence from ingested prey cells (Holmes et al., 1967). This could also be the case for heterotrophs such as dinoflagellates, ciliates, and foraminifera that retain chloroplasts from their prey through kleptoplasty (Hansen et al., 2013; Stoecker et al., 2009). This is not an issue for pallium‐feeding dinoflagellates such as *Protoperidinium* that undergo external digestion of prey, with extracellular breakdown of chlorophyll, but would apply to grazers such as *P. kofoidii*, *Noctiluca scintillans*, *Gyrodinium*, and other heterotrophic dinoflagellates, as well as other protists that ingest whole prey. Considering the importance of mixotrophs in the upper ocean (Jeong et al., 2015, 2021; Mansour & Anestis, 2021), the presence of fluorescently active and photosynthetically active chloroplasts in heterotrophic plankton will increasingly impact the biological interpretation of chlorophyll measurements and the modeling of ocean primary production.

### Final thoughts: Bioluminescence properties of *Polykrikos kofoidii* infer nuanced differences across dinoflagellates

The bioluminescence of *Polykrikos kofoidii*, a globally distributed heterotrophic dinoflagellate, is characterized by unique features not present in other bioluminescent dinoflagellates. Although its bioluminescence and fluorescence emission spectra were similar to those of other luminescent dinoflagellates, reflecting a similar luminescent chemical family, mechanically stimulated flashes have slower kinetics and dimmer intensity. Surprisingly, the distribution of cellular autofluorescence indicative of luciferin was diffusely spread throughout the cytoplasm, unlike all other studied dinoflagellates in which luciferin is localized solely within scintillons with fluorescence (and bioluminescence) originating from punctate sources. The ecological relevance of the differences in flash kinetics and spatial emission remains to be determined, especially in terms of possible alternative functions associated with the light production. The luciferase of *P. kofoidii* was similar to that of luminescent dinoflagellates, containing three catalytic domains but with a different N‐terminal domain, also observed in an undescribed *Protoperidinium* species. *Polykrikos kofoidii* potentially contains a hybrid luciferase‐luciferin binding protein gene similar to that of *Noctiluca scintillans*, which is considered a basal dinoflagellate. These findings emphasize the value of studying additional dinoflagellate taxa, especially heterotrophs, to gain a better understanding of the evolution of luminescent chemistry and genetics.

## AUTHOR CONTRIBUTIONS

**Michael I. Latz:** Conceptualization (equal); data curation (equal); formal analysis (equal); investigation (equal); methodology (equal); project administration (equal); resources (equal); software (equal); supervision (equal); validation (equal); visualization (equal); writing – original draft (lead); writing – review and editing (equal). **Dimitri D. Deheyn:** Methodology (supporting); resources (supporting); writing – review and editing (supporting). **Brittany N. Sprecher:** Conceptualization (equal); data curation (equal); formal analysis (equal); funding acquisition (lead); investigation (equal); methodology (equal); project administration (equal); resources (equal); software (equal); supervision (equal); validation (equal); visualization (equal); writing – original draft (equal); writing – review and editing (equal).

## Supporting information


**Figure S1** Light microscope images of the fluorescence of heterotrophic dinoflagellates obtained with the PARISS hyperspectral imaging system.
**Figure S2**. Bayesian phylogenetic tree for luciferase catalytic domains with posterior probabilities.
**Figure S3**. Amino acid alignment in Geneious (v 9.1.8) of the four separated LBP internal repeats (D1‐D4) for *Noctiluca scintillans lcf‐lbp* gene (NsLCF) GenBank Accession AED02505.1, *Polykrikos kofoidii lcf* N‐terminal *lbp‐*like motif (PkLCF), *Protoperidinium* sp. 1 *lcf* N‐terminal *lbp‐*like motif (PsLCF), and *Lingulaulax polyedra lbp* (LpLBP) GenBank Accession AAA29164.1. Colored amino acids have a coverage of >50% across all sequences.
**Table S1**. Information about transcriptomes used in this study, including species name, the assembly name, and published reference.

## Data Availability

All 18S and ITS sequences are available on the GenBank database (accession numbers PX496324‐496329). All gene alignment files can be accessed at figshare under doi:10.6084/m9.figshare.30724736.
