# Supplementary material for: Bioluminescence of the heterotrophic dinoflagellate Polykrikos kofoidii Chatton 1914 (Dinophyceae)
Source: J Phycol. 2026 Mar 31;62(2):666–88. doi: 10.1111/jpy.70157 (PMC13103706; doi:10.1111/jpy.70157)
Supplement: Supplementary file 1 — Figure S1 Light microscope images of the fluorescence of heterotrophic dinoflagellates obtained with the PARISS hyperspectral imaging system. Figure S2. Bayesian phylogenetic tree for luciferase catalytic domains with posterior probabilities. Figure S3. Amino acid alignment in Geneious (v 9.1.8) of the four separated LBP internal repeats (D1‐D4) for Noctiluca scintillans lcf‐lbp gene (NsLCF) GenBank Accession AED02505.1, Polykrikos kofoidii lcf N‐terminal lbp‐like motif (PkLCF), Protoperidinium sp. 1 lcf N‐terminal lbp‐like motif (PsLCF), and Lingulaulax polyedra lbp (LpLBP) GenBank Accession AAA29164.1. Colored amino acids have a coverage of >50% across all sequences. Table S1. Information about transcriptomes used in this study, including species name, the assembly name, and published reference. [file JPY-62-666-s001.pdf]

Bioluminescence of the heterotrophic dinoflagellate *Polykrikos kofoidii* Chatton 1914 (Dinophyceae): Supplementary information

Michael I. Latz*+

Dimitri D. Deheyn

Scripps Institution of Oceanography, University of California San Diego, La Jolla, California 92093-0202

Brittany N. Sprecher*

Department of Neurosciences, University of California San Diego School of Medicine, La Jolla, California 92093

*Share equal authorship

+Corresponding author: mlatz@ucsd.

Table S1. Information about transcriptomes used in this study, including species name, the assembly name, and published reference. BUSCO estimated completeness is based on the Alveolata database.

Species	Sequencing Identifier	BUSCO Completeness (Percent)	Reference
Alexandrium andersonii	MMETSP1436	45.6	Johnson et al., 2019
Alexandrium catenella	MMETSP0790	78.4	Johnson et al., 2019
Alexandrium fundyense	MMETSP0196; MMETSP0197; MMETSP0347	39.8	Johnson et al., 2019
Alexandrium margalefii	MMETSP0661	84.4	Johnson et al., 2019
Alexandrium minutum	MMETSP0328	37.4	Johnson et al., 2019
Alexandrium monilatum	MMETSP0097; MMETSP0096; MMETSP0095; MMETSP0093	90.6	Johnson et al., 2019
Alexandrium tamarense	MMETSP0384; MMETSP0382; MMETSP0380; MMETSP0378	93.6	Johnson et al., 2019
Gonyaulax spinifera	MMETSP1439	69	Johnson et al., 2019
Noctiluca scintillans	MMETSP0253	92.4	Johnson et al., 2019
Noctiluca scintillans	Nsc-QI	31	Cooney et al., 2024
Polykrikos kofoidii	PkFC1-7; PkQI8	74.9 (combined)	Cooney et al., 2023
Polykrikos kofoidii	SRR11994206	97.1	Jeong et al., 2021
Polykrikos lebouriae		24	Gavelis et al., 2015
Protoceratium reticulatum	MMETSP0228	83	Johnson et al., 2019
Protoceratium reticulatum	Pre-FC	14	Cooney et al., 2024
Protoperidinium conicum	Pco-FC1; Pco-QI2	38	Cooney et al., 2024
Protoperidinium depressum	Pde-GA	42.1	Cooney et al., 2024
Protoperidinium pellucidum	Ppe-QI2	32.7	Cooney et al., 2024
Protoperidinium sp. 1	Ps1-FC1; Ps1-FC2; Ps1-QI3; Ps1-QI4	36.3	Cooney et al., 2024
Protoperidinium sp. 2	Ps2-Q	23.4	Cooney et al., 2024
Pyrocystis lunula	MMETSP0229	1.2	Johnson et al., 2019
Pyrodinium bahamense	MMETSP0796	82.5	Johnson et al., 2019
Tripos fusus	MMETSP1074; MMETSP1075	81.3	Johnson et al., 2019

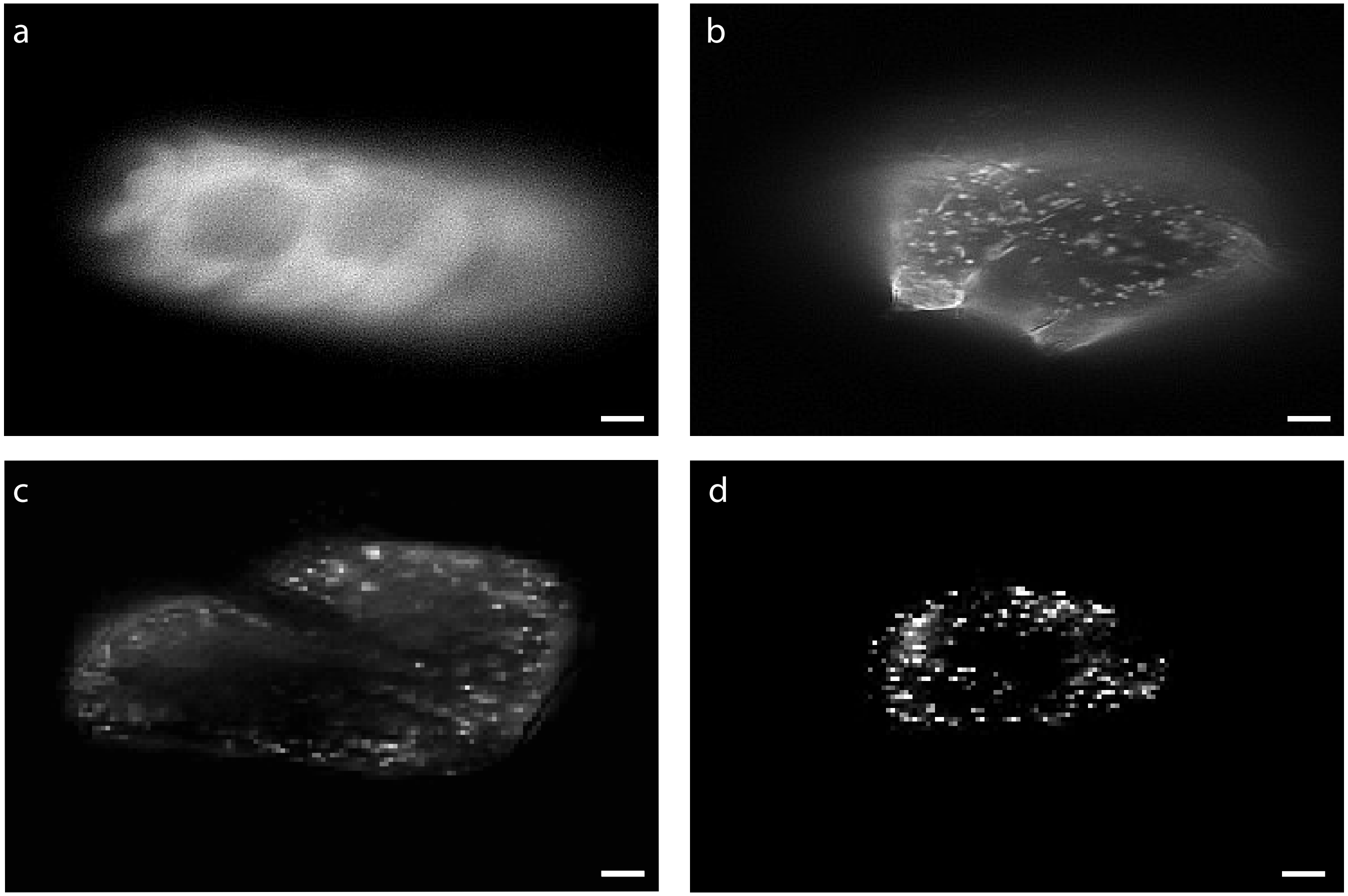

Figure S1. Light microscope images of the fluorescence of heterotrophic dinoflagellates obtained with the PARISS hyperspectral imaging system. (A) *Polykrikos kofoidii*; (B) *Protoperidinium divergens*; (C) *Pr. oblongum*; (D) *Pr. steinii.* Scale bars represent 10 µm.

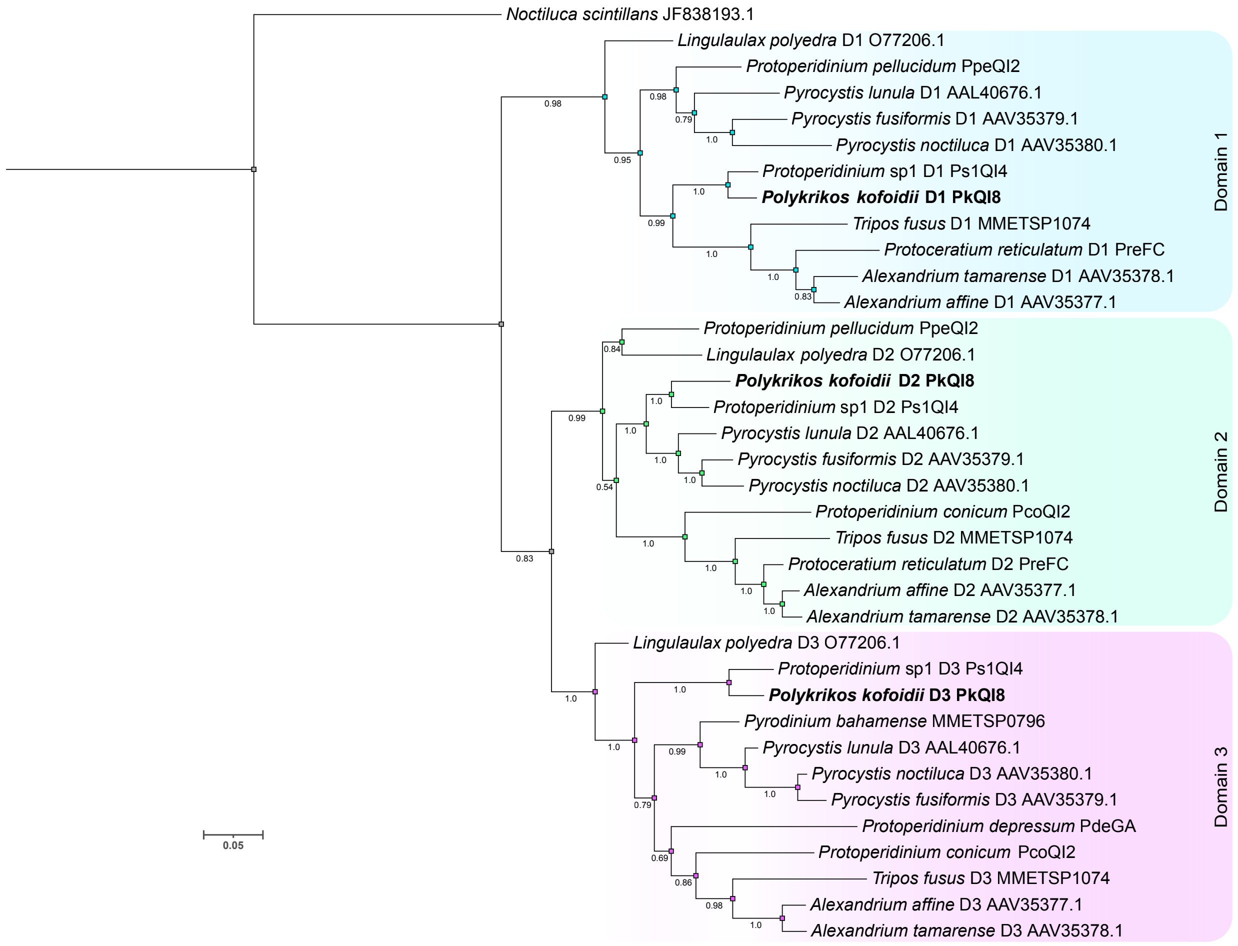

Figure S2. Bayesian phylogenetic tree for luciferase catalytic domains with posterior probabilities. Luciferase catalytic domain 1 is highlighted in blue (D1), domain 2 in green (D2), and domain 3 in purple (D3). GenBank accession numbers or transcriptome names are shown at the end of each species label, and *Polykrikos kofoidii* sequences are labeled in bold.

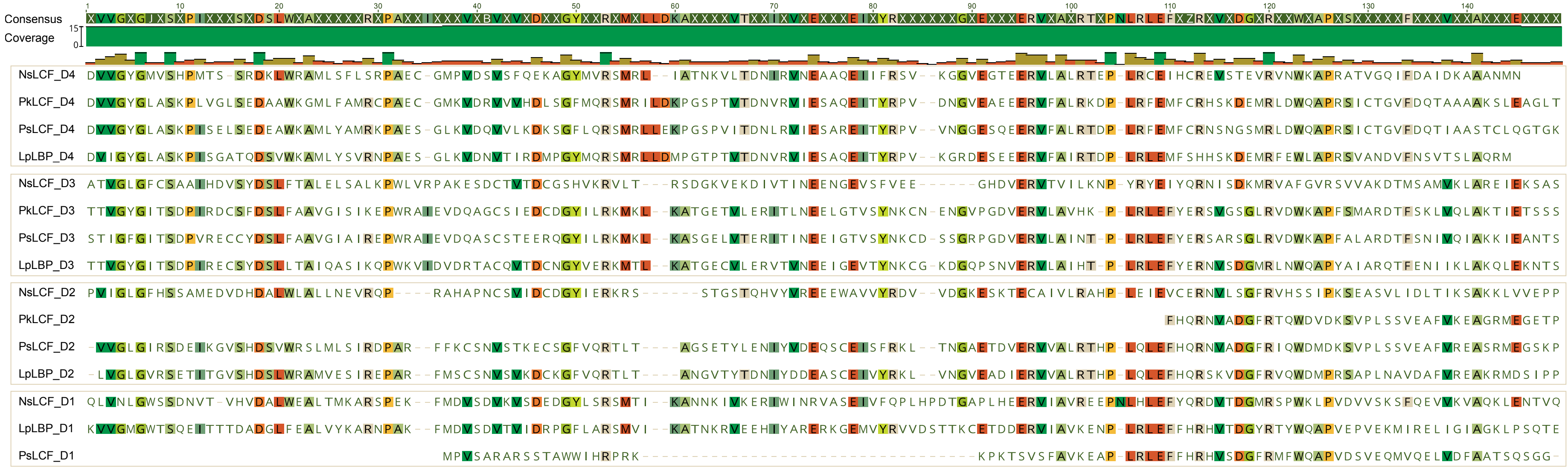

Figure S3. Amino acid alignment in Geneious (v 9.1.8) of the four separated LBP internal repeats (D1-D4) for *Noctiluca scintillans lcf-lbp* gene (NsLCF) GenBank Accession AED02505.1, *Polykrikos kofoidii lcf* N-terminal *lbp*-like motif (PkLCF), *Protoperidinium* sp. 1 *lcf* N-terminal *lbp*-like motif (PsLCF), and *Lingulaulax polyedra lbp* (LpLBP) GenBank Accession AAA29164.1. Colored amino acids have a coverage of >50% across all sequences.

REFERENCES

Cooney, E. C., Holt, C. C., Jacko-Reynolds, V. K. L., Leander, B. S., & Keeling, P. J. (2023). Photosystems in the eye-like organelles of heterotrophic warnowiid dinoflagellates. *Current Biology*, *33*, 4252.

Cooney, E. C., Holt, C. C., Hehenberger, E., Adams, J. A., Leander, B. S., & Keeling, P. J. (2024). Investigation of heterotrophs reveals new insights in dinoflagellate evolution. *Molecular Phylogenetics and Evolution*, *196*, 108086.

Gavelis, G. S., White, R. A., Suttle, C. A., Keeling, P. J., & Leander, B. S. (2015). Single-cell transcriptomics using spliced leader PCR: Evidence for multiple losses of photosynthesis in polykrikoid dinoflagellates. *BMC Genomics*, *16*, 528.

Jeong, H. J., Kang, H. C., Lim, A. S., Jang, S. H., Lee, K., Lee, S. Y., Ok, J. H., You, J. H., Kim, J. H., Lee, K. H., Park, S. A., Eom, S. H., Yoo, Y. D., & Kim, K. Y. (2021). Feeding diverse prey as an excellent strategy of mixotrophic dinoflagellates for global dominance. *Science Advances*, *7*, eabe4214.

Johnson, L. K., Alexander, H., & Brown, C. T. (2019). Re-assembly, quality evaluation, and annotation of 678 microbial eukaryotic reference transcriptomes. *GigaScience*, *8*, gly158.
